# The Use of Yeast Mixed Cultures for Deacidification and Improvement of the Composition of Cold Climate Grape Wines

**DOI:** 10.3390/molecules26092628

**Published:** 2021-04-30

**Authors:** Monika Cioch-Skoneczny, Michał Grabowski, Paweł Satora, Szymon Skoneczny, Krystian Klimczak

**Affiliations:** 1 Department of Fermentation Technology and Microbiology, Faculty of Food Technology, University of Agriculture in Krakow, ul. Balicka 122, 30-149 Krakow, Poland; michalgrabowski301@gmail.com (M.G.); pawel.satora@urk.edu.pl (P.S.); leos4815@gmail.com (K.K.); 2 Department of Chemical and Process Engineering, Faculty of Chemical Engineering and Technology, Cracow University of Technology, Warszawska 24, 31-155 Krakow, Poland; szymon.skoneczny@pk.edu.pl

**Keywords:** non-*Saccharomyces* yeast, mixed cultures, biological deacidification, volatile compounds, organic acids

## Abstract

Interest in the use of non-*Saccharomyces* yeast in mixed cultures is increasing due to the perceived improvement in the quality and complexity of the resulting wines. The aim of the study was to determine the ability of monocultures and mixed yeast cultures for deacidification and improvement of the composition of cold climate grape wines. Fermentation of grape musts with increased total acidity was carried out with the use of monocultures of *Saccharomyces cerevisiae* MH020215 (Sc), *Zygosaccharomyces bailii* 749 (Zb) and *Metschnikowia pulcherrima* MG970690 (Mp), and their mixed cultures, inoculated simultaneously and sequentially. Oenological parameters, organic acids and volatile compounds profiles of obtained wines were characterized. The fermentation kinetics and analytical profiles of the obtained wines showed that the use of mixed yeast cultures contributed to the reduction of volatile acidity and acetic acid content in the wines, as well as obtaining a favorable aromatic profile of the wines. The dominant higher alcohols in all wines were 2-methyl-1-propanol, 3-methyl-1-butanol and 2-methyl-1-butanol. Significantly higher amounts of the first two compounds were found in wines obtained with *M. pulcherrima* MG070690, both in monoculture and in mixed cultures. The monocultures of *M. pulcherrima* MG070690 (Mp) compared with *Z. bailli* 749 (Zb) synthesized higher levels of esters in wines, including ethyl acetate, ethyl propionate, isobutyl acetate, ethyl pyroracemate and isoamyl acetate.

## 1. Introduction

Organic acids present in grapes are the main source of wine acidity. The chemical composition of the grapes affects the composition of juice, and finally-the quality of the product. One of the key reasons for excessive wine acidity is l-malic acid, the concentration of which in grapes varies from 1 to 16 g/L, depending on the climate, region, season, and grapevine variety [1,2]. l-malic acid is accumulated mainly in the peel of the grapes and its amount is much lower in the flesh and the grape juice. This tendency may change during the ripening of the berries and due to technical treatments [3,4]. l-Malic and l-tartaric acids usually constitute more than 90% of the total acidity of the grapes [5]. Content of l-malic acid in *Vitis vinifera* berries from cold climate wine regions are usually higher (15–16 g/L), as the low temperature favours the acid respiration process, unlike warm climate regions. l-Malic acid not only contributes to the increase in wine acidity but also serves as a substrate for the growth of bacteria and yeast, the presence of which leads to undesirable changes in the product after its bottling [6]. Therefore, removing excess l-malic acid is important to ensure physical, biochemical and microbiological stability, which in turn contributes to the quality improvement of the produced wine. A natural method for obtaining less sour wine is biological deacidification, which can be achieved through malolactic fermentation (MLF) or malo-ethanolic fermentation (MEF). During MLF, lactic acid bacteria, inter alia *Oenococcus oeni*, convert malic acid to lactate and CO_2_, while MEF is mainly performed by yeasts, such as *Schizosaccharomyces pombe* [1,7,8,9]. Malolactic fermentation is a traditional wine deacidification method. However, due to problems associated (production of biogenic amines, ethyl carbamate, changes in the organoleptic characteristics of wine), alternative techniques are sought to reduce the acidity of wines. Deacidification using yeast strains has many benefits. It contributes to the degradation of l-malic acid but also reduces the risk of the formation of undesired compounds. Moreover, simultaneous alcoholic fermentation and deacidification prevent wine spoilage due to oxidation and inhibit the growth of harmful microorganisms. Therefore, the deacidification of wines with yeast offers the possibility of introducing better strategies to optimize the vinification process.

A few decades ago, the term “non-*Saccharomyces*” was usually associated with wine spoilage, mainly due to the increase in volatile acidity or formation of undesirable compounds such as volatile phenols (for which representatives of *Brettanomyces* genus were responsible) [10,11]. However, over the past years, many studies have demonstrated the metabolic/enzymatic potential of certain non-*Saccharomyces* yeasts, and their role in improving some technological and sensory aspects of wine [12,13,14]. The application of non-*Saccharomyces* yeasts has become a trend in the modern wine industry. Currently, several species of non-*Saccharomyces* yeast are used on an industrial scale, including *Torulaspora delbrueckii*, *Lachancea thermotolerans*, *Metschnikowia pulcherrima* and *Pichia kluyveri*. The advantage of their application is, among others, modification of the aroma [15,16], control of acidity [17], improvement of color and mouthfeel properties [18], as well as decreasing the ethanol content in wines [19,20]. In recent years, the number of studies using non-*Saccharomyces* yeast has increased significantly. However, still many species have not yet been well investigated, including yeasts of *Candida*, *Hanseniaspora*/*Kloeckera*, *Metschnikowia*, *Pichia*, *Torulaspora* and *Zygosaccharomyces* genera.

The aim of the study was to determine the ability of yeast monocultures and mixed cultures to reduce the total acidity and improve the chemical composition of cold climate grape wines. Fermentation of grape musts with increased total acidity was carried out with the use of monocultures of *Saccharomyces cerevisiae* MH020215 (Sc), *Zygosaccharomyces bailii* 749 (Zb) and *Metschnikowia pulcherrima* MG970690 (Mp), and their mixed cultures, inoculated simultaneously and sequentially. Oenological parameters, organic acids and volatile compounds profiles of obtained wines were characterized. The non-*Saccharomyces* strains used in this research, although periodically mentioned in scientific literature as wine-related yeasts, have not been thoroughly characterized in terms of their deacidification properties. There are studies concerning the use of *Z. bailli* yeast to deacidify wines [21,22]. However, little information is available on deacidification of cold climate wines using *M. pulcherrima* and *Z. bailli*. Hence the idea to conduct new research to enrich this area of knowledge.

## 2. Results and Discussion

### 2.1. The Dynamics of the Fermentation Process

**Figure 1:**
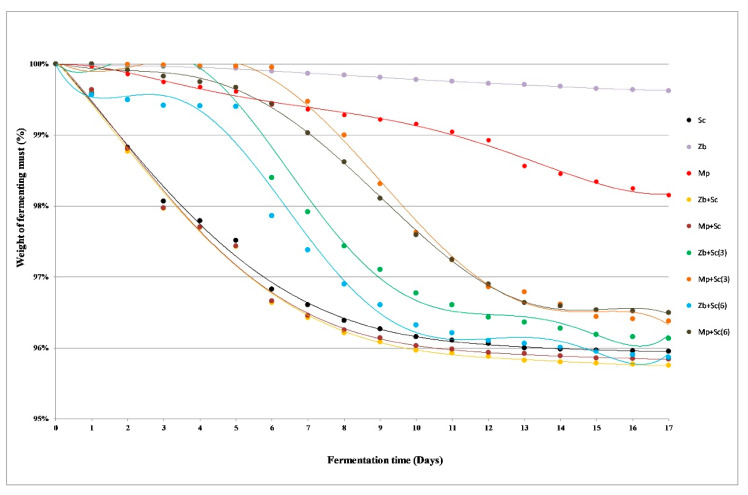
The fermentation dynamics of grape musts (abbreviations are described in Section 3.1.2. Grape Musts Fermentation).

Ethyl alcohol, carbon dioxide and a wide range of by-products are produced as a result of carbohydrate metabolism during the must fermentation. The weight losses of fermenting batches are the effect of carbon dioxide release [23]. The samples inoculated with *S. cerevisiae* MH020215 (Sc) showed clearly the best fermentation dynamics among analysed monocultures (Figure 1). The weight losses of the batches were noticeable from the first day, although most of the carbon dioxide was released between the third and seventh day of the fermentation. A reduction in the fermentation rate was observed from around the 10th day. The small mass loss of the batches inoculated with *Z. bailii* 749 monoculture confirmed that this strain exhibits low ethanol fermentation abilities (Figure 1). The fermentation activity of *Z. bailli* yeasts is much lower than that of the *S. cerevisiae*, which is related to the low growth rate [24]. *Z. bailii* however, has a much higher tolerance for ethanol and some acids, therefore fermentation proceeds longer, and the cells do not degrade [25]. *M. pulcherrima* yeast are the natural microbiota of grapes. It participates in the initial stages of spontaneous fermentation, accounting up to 20% of microorganisms involved [26]. In the case of grape musts inoculated with *M. pulcherrima* MG970690 (Mp), the significant weight loss of the samples was observed (Figure 1). The fermentation dynamics of simultaneous fermentation of *Z. bailii* 749 and *S. cerevisiae* MH020215 (Zb+Sc), as well as *M. pulcherrima* MG970690 and *S. cerevisiae* MH020215 (Mp+Sc) proceeded similarly. Weight losses of the fermented batches were analogous as in *S. cerevisiae* MH020215 (Sc) monoculture (Figure 1).

Non-*Saccharomyces* yeast gain dominance during the initial stages of fermentation, which causes an increase in their amount. They are unable to produce high concentrations of alcohol, but they impart the desired sensory characteristics to the wines [27]. A rapid weight loss of the batches in sequential fermentation samples occurred after adding *S. cerevisiae* MH020215 yeast (Figure 1). For all trials inoculated on the third and sixth day, this phenomenon was observed on the next day of the process. The mixed cultures with *Z. bailli* 749 were characterized by a slightly better degree of attenuation (Figure 1).

### 2.2. Oenological Parameters

The term Free Amino Nitrogen (FAN) describes the content of amino acids and short-chain proteins in the must, which can be used by yeast as a building material. This parameter is a measure of the free nitrogen that is assimilable by the yeast cells. FAN content in wines depends on many factors, such as grapevine variety, soil quality, winegrowing practices and the usage of plant protection products [28]. The main sources of nitrogen needed for the growth of microorganisms during the fermentation process are free amino acids and ammonium ions. Literature data indicates that some juices may contain insufficient amounts of these components, which are required to ensure optimal growth of microorganisms and proper fermentation. Additionally, nitrogen requirement increases in must with the sugar concentration [7,29]. The content of free amino nitrogen in the grape must was 106 mg/L (Table 1). As expected, the lowest FAN values were found in wines subjected to simultaneous and sequential fermentation, as well as inoculated with yeast *S. cerevisiae* MH020215. The *Z. bailli* 749 (Zb) and *M. pulcherrima* MG970690 (Mp) monocultures utilized much lower amounts of FAN during the fermentation process (Table 2). This suggests that these species require less nitrogen compounds.

All produced wines were subjected to chemical analysis to determine the content of alcohol, real extract and free amino nitrogen. The wine obtained by fermentation with the monoculture of *S. cerevisiae* MH020215 (Sc) (Table 2) was characterized by the highest ethyl alcohol concentration. Comparable values were acquired for samples inoculated simultaneously (Zb+Sc, Mp+Sc) (Table 2). As is known, *S. cerevisiae* dominates in the later stages of fermentation. The high initial biomass of non-*Saccharomyces* yeast gradually decreases, and often even completely disappears. Such a high ethanol content in the samples inoculated simultaneously was caused by the presence of *S. cerevisiae* in the fermenting must. Relatively high ethanol concentrations were also found in the case of sequential fermentation. Wines obtained with *Z. bailli* 749 (Zb) monoculture were characterized by low alcohol content, which confirmed also fermentation dynamics (Table 2, Figure 1). According to the literature, *Z. bailli* produces small amounts of ethanol. Its content in obtained wine was only 4 g/L. This result was over 60% lower than that of *S. cerevisiae* [30]. *M. pulcherrima* MG970690 (Mp) monoculture produced approximately 7% alcohol in wine (Table 2). These yeasts demonstrated superior fermentation capacity as compared to *Z. bailii*. During spontaneous fermentation they persist in the initial stages of the process, afterwards, the dominance of *S. cerevisiae* strains occurs. However, it has been shown, that the concentration of alcohol produced by *M. pulcherrima* yeast can reach even 10% [31].

The real extract consists of compounds remaining after simple distillation and it is a good indicator of sugars content and the degree of their utilization. The content of the real extract is related to the concentration of ethyl alcohol and residual sugars. The higher concentration of ethanol is produced, the less sugars remain after fermentation, and therefore the real extract is lower [32]. The value of this parameter in the analysed wines ranged from 20.6–25.8 g/L, except wines obtained with *Z. bailii* 749 (Zb) and *M. pulcherrima* MG970690 (Mp) monocultures (Table 2). Despite different methods of inoculation, *S. cerevisiae* MH020215 utilizes similar amounts of sugar. The addition of other cultures, such as *Z. bailii* 749 or *M. pulcherrima* MG970690 for deacidification does not reduce fermentation efficiency. In some cases, it is even slightly improved [14]. The yeast *Z. bailli* 749 showed very favorable deacidifying properties. Sequential fermentation resulted in a greater reduction of total acidity in the obtained wines, as compared to the simultaneous fermentation. On the other hand, *M. pulcherrima* MG970690 yeast did not deacidify the wines. However, the monoculture showed good fermentation properties (Table 2).

### 2.3. Total Acidity and Organic Acids

Total acidity is one of the basic parameters determining the winemaking process and affecting wine quality, as well as balanced taste. In the finished product, it should not exceed 10 g/L [33]. The initial acidity of the must before the fermentation was adjusted for every batch to 10 g/L, expressed as a malic acid concentration (g malic acid/L), (Table 1). *Z. bailii* 749 showed very favourable deacidification properties (Table 2). In contrast to *S. cerevisiae*, *Z. bailii* cells are able to assimilate l-malic acid in a relatively short time, but they require a carbon source in the form of sugars for the process [34]. Sequential fermentation resulted in a greater reduction in the acidity of obtained wines, compared to the simultaneous inoculation (Table 2). Based on the obtained results, it can be supposed that intensively growing *S. cerevisiae* MH020215 cells use the sugar from the must for the alcoholic fermentation. It causes depletion in carbon source for *Z. bailii* 749 and stops the acids decomposition. Delayed inoculation with *S. cerevisiae* MH020215 (Zb+Sc(3) and Zb+Sc(6)) seems to have a positive effect on the deacidification capacity of the mixed culture. However, according to the literature, the extended period of *Z. bailii* activity may cause the formation of acetic acid, which negatively affects the sensory characteristics of the wine [14]. *S. cerevisiae* yeasts individually are able to break down the excess of this compound in amounts from 3 to even 45%. However, this ability primarily depends on the varied levels of expression of the gene encoding the malic enzyme, which in turn is related to several environmental factors [3]. The analysis of the acidity of wines produced using mixed cultures revealed a clear relationship that later inoculation of *S. cerevisiae* MH020215 decreases the value of this parameter in the finished product (Table 2).

Volatile acidity is another factor determining the quality of the wine. It is usually expressed as acetic acid concentration in the beverage. This compound is formed as a result of the conversion of ethyl alcohol to acetaldehyde, followed by its transformation with the participation of the aldehyde dehydrogenase. Acetic acid is responsible for 90% of the volatile acidity of wines while the remaining fatty acids (propanoic and butanoic acid) are present in small amounts [7]. Acetic acid is usually formed in amounts of 0.2–0.8 g/L [33]. The levels of volatile acidity in analysed wines were low (Table 2). The highest volatile acidity was found in the samples fermented by *M. pulcherrima* MG970690 monoculture (Mp)-0.3 g/L. A slightly lower value was found in wines obtained with *Z. bailii* 749 (Zb) monoculture (Table 2). Some non-*Saccharomyces* yeast (such as *Hanseniaspora*, *Zygosaccharomyces*, *Schizosaccharomyces*) are producers of a considerable amount of acetic acid [35,36,37]. However, most non-*Saccharomyces* yeast are not capable of producing high amounts of volatile acids. Their content rarely exceeds 0.2 g/L after fermentation with *M. pulcherrima* or *Z. bailli* [38]. Similar levels of the analysed parameter were achieved both in simultaneous and sequential fermentations, ranging from 0.16 to 0.19 g/L (Table 2). Also, relatively low volatile acidity values were obtained by Comitini et al. [39] who used mixed cultures of *M. pulcherrima* and *S. cerevisiae* in the fermentation (0.30–0.34 g/L).

The main organic acids found in grapes are l-tartaric and l-malic acids. Although these compounds have a similar chemical structure, they are synthesized from glucose in fruits using different metabolic pathways. l-malic acid is formed in the reaction of glycolysis and the tricarboxylic acid (TCA) cycle, while ascorbate is an intermediate product of l-tartaric acid biosynthesis. Tartaric acid is usually present in grapes in concentrations from 5 to 10 g/L, while the content of l-malic acid in ripe grapes ranges most often between 2 and 6.5 g/L [8]. In fresh grape must used for fermentation, 2.54 g/L tartaric acid was detected (Table 1). Tartaric acid is not metabolized in the fermentation process, so its amounts are usually constant [40]. This has been confirmed in our research (Table 3). Tartaric acid is very stable, but in rare cases, it may decrease as a result of the precipitation of potassium tartrate [41]. Vonach et al. [42] indicate the presence of this component in wine in the amount of 1.06–1.68 g/L. Other literature sources report that its content can be as high as 4.55 g/L [43]. On the other hand, in Slovak wines, the concentration of l-tartaric acid ranged from 0.95 to 2.58 g/L. Dobrowolska-Iwanek et al. [44] noted over 10 times lower tartaric acid content in wine obtained from the Leon Millot variety (0.224 g/L) compared to our research. Similarly, low values of the analyzed component were found in the Rondo and Regent wines. In turn, white wines were characterized by higher tartaric acid content.

Cold climate wines are characterized by a higher content of l-malic acid compared to tartaric acid. In turn, the reduction of malic acid content takes place during the malolactic fermentation process, as a result of which malic acid is converted into lactic acid [40]. The greatest decrease in malic acid was found in the wines inoculated sequentially (Zb+Sc(3), Mp+Sc(3), Zb+Sc(6), Mp+Sc(6)) (Table 3). In the experiments carried out by Soyer et al. [2], the content of l-malic acid in grapes ranged from 1.43 to 3.40 g/L. Much lower amounts of acid were found in grapevines from Chile (0.39–1.8 g/L) [45]. On the other hand, the l-malic acid content in cold climate wines reaches even 6.07 g/L [46]. Research by Dobrowolska-Iwanek et al. [44] showed a different ratio of tartaric and malic acid in cold climate wines. In contrast to white wines, in the case of wines obtained from the Leon Millot, Merechal Foch and Regent varieties, a greater amount of tartaric acid was observed compared to l-malic acid. It is worth mentioning that by the decision of the Council of Europe of December 20, 2005, the territory of Poland was included in zone A of viticulture in Europe. Lower temperatures result in lower sugar content in the fruit and increased acidity. Nevertheless, the chemical composition of the grapes grown in the cold climate zone allows for the achievement of high-quality wines that can be perceived as more harmonized and fresh, thanks to the different proportion of acids and sugars contained in grapes.

Despite the fact that citric acid is present in trace amounts in grapes (0.5–1 g/L), it plays an important role in biochemical and metabolic processes (Krebs cycle). l-Tartaric acid is resistant to degradation by microorganisms during the fermentation process, while malic and citric acids can be partially metabolized by yeast and bacteria, which results in the reduction of wine acidity [47,48]. In all analysed wines, citric acid was found at a similar level (Table 3).

The concentration of lactic acid in wine is usually between 0.2–0.4 g/L, although some sources mention as much as 6 g/L [49]. This component was not noted in most of the analysed wines. In wines obtained with the *Z. bailli* 749 (Zb) and *M. pulcherrima* MG970690 (Mp) monocultures, the highest concentration of this component was found (2.30 and 1.61 g/L). Some yeasts are able to produce malic and lactic acid affecting the pH of wines. Some non-*Saccharomyces* yeasts can produce it in high concentration, even in oenological conditions at a variable range of pH [8,50,51].

Succinic acid was found in all analysed wines (Table 3). The succinic acid concentration in wine is at the level of 0.5–1.5 g/L. Values above 3.0 g/L may be present in some red wines and may not necessarily negatively affect the taste of the wine [52]. The *Z. bailli* strain is known for the high production of succinic and acetic acid in wine [53]. On the other hand, the use of mixed cultures of *M. pulcherrima* MG970690 and *S. cerevisiae* MH020215 (Mp+Sc, Mp+Sc (3), Mp+Sc (6)), both as a result of simultaneous and sequential fermentation, resulted in obtaining a lower level of acetic acid in wines, in comparison with the monoculture of *M. pulcherrima* MG970690 (Table 3). Low acetic acid production in wines with the participation of non-*Saccharomyces* yeast in mixed cultures with *S. cerevisiae* was also demonstrated in the studies of other authors [16,31,39,54,55].

### 2.4. Volatile Compounds

Wine is one of the products with the most complex flavour profile. The aroma is influenced by the variety of grapevine used, the conditions of cultivation and harvest, while the characteristic bouquet is the result of the fermentation process, maturation and aging process of the wine. Most of the aroma compounds that are released into wine from grapes are present in the form of odourless glycosides [56].

Table 4 characterizes the components present in fresh grape must. It is known that higher alcohols are produced in the wine fermentation process, affecting the sensory profile of the drink [57]. However, literature sources also indicate trace amounts of these components in grapes, mainly in skins [58]. In fresh grape must, small amounts were noted, including 1-hexanol, 3-hexen-1-ol, 1-undecanol and 1-nonanol, as well as other compounds, including acetals (Table 4). The concentration of these compounds varies depending on the grapevine variety, climatic and agrotechnical factors [48].

Table 5 present the volatile components of obtained wines. One of the main group of compounds synthesized by yeast are higher alcohols, also known as fusels. Their molecules contain more than two carbon atoms and are characterized by higher molecular mass, as well as boiling point in comparison to ethanol. They are produced during the fermentation process, and their concentration reaches approx. 150–550 mg/L. In chemical terms, they can be divided into aliphatic and aromatic alcohols. The first group consists of inter alia propanol, isobutanol and amyl alcohols. The second includes: 2-phenylethanol, tyrosol or tryptophol. Fusel alcohols have an intense aroma that plays an important role in forming a wine bouquet. At low concentrations (below 300 mg/L) they positively affect its aroma whereas higher contents mask the proper aroma of the beverage [33]. 2-methyl-1-propanol, 3-methyl-1-butanol and 2-methyl-1-butanol were prevailing higher alcohols in all of the samples (Table 5). In the case of the two first listed components, significantly higher concentrations were found in wines inoculated sequentially (additional inoculation on the sixth day of fermentation). *M. pulcherrima* MG970690 (Mp) monoculture produced higher amounts of these compounds in the analysed wines, as compared to *S. cerevisiae* MH020215 (Sc). Obtained data do not match the results of other authors, who claim that non-*Saccharomyces* yeasts are responsible for the formation of lower concentrations of higher alcohols (*n*-propanol, isobutanol, amyl alcohols) in relation to *S. cerevisiae* strains [59,60]. However, the formation of these components is assessed based on a number of factors, including oxygen content, sugar content in grapes, temperature and maceration time [4,48,61]. The beneficial effect of non-*Saccharomyces* has been repeatedly demonstrated in mixed culture with wine strains. Wines obtained with the participation of *S. cerevisiae*, *T. delbrueckii* and *M. pulcherrima* were characterized by a high content of higher alcohols [39]. A combination of microorganisms can be used for obtaining unique wine aromas that could not be obtained with monocultures. Non-*Saccharomyces* yeast produced higher levels of 1-decanol, 1-undecanol, 2-dodecanol, 1-dodecanol and 1-hexanol in wines (Table 5). 3-methylpentanol and isohexanol were found only in wines inoculated with *S. cerevisiae* MH020215 yeast. Phenylethanol was detected in significant amounts in wines that were at least partially inoculated with *S. cerevisiae* MH020215. Significantly lower concentrations were found in samples fermented with *M. pulcherrima* MG970690 (Mp) and *Z. bailii* 749 (Zb) monocultures (Table 5). Escribano et al. [62] determined the fermentation capacity and aroma formation for several non-*Saccharomyces* yeasts. They suggested that *M. pulcherrima* owns good properties for wine fermentation and creates high concentrations of 2-phenylethyl alcohol and 2-phenylethyl acetate [63].

Esters are a broad group of by-products found in fermented beverages. They can be formed as a result of chemical condensation of carboxylic acids and alcohols, however mainly are the products of yeast metabolism. The enzymatic synthesis of esters is catalysed by many microbial enzymes (esterases, lipases), including acetyltransferases. Alcohol and acetyl coenzyme A are the substrates for this reaction. Currently, approximately 160 specific esters have been recognized. Depending on the substrates from which they are formed, volatile and non-volatile esters can be distinguished. Compounds belonging to the first group positively affect wine bouquet, while those from the second group act as a component of taste [64]. Among all identified esters in the tested samples, ethyl acetate was found in the highest quantities. Substantial amounts of this compound were identified in wines inoculated with *M. pulcherrima* MG970690 (Mp) and *S. cerevisiae* MH020215 (Sc) monocultures. Lower concentrations were found in samples inoculated with *Z. bailii* 749 (Zb) monoculture (Table 5). Both simultaneous and sequential fermentation increased quantities of ethyl acetate in the analysed wines compared to monocultures (Table 4). Similar results were obtained by Comitini et al. [39], using a mixed culture of *S. cerevisiae* and *M. pulcherrima* for the fermentation of the grape must. Yeast belonging to the *Saccharomyces* genus synthesizes mainly ethyl and acetate esters. Studies show that small quantities of ethyl acetate (50–80 mg/L) have a positive effect on the quality of the drink, whereas too high concentrations can lead to abnormal aftertaste [4]. Content of isoamyl acetate, 2-phenylethyl acetate and isobutyl acetate was several dozen, or even several hundred times lower than that of ethyl acetate (Table 5). According to the literature data, the use of mixed strains of *M. pulcherrima* and *S. cerevisiae* yeast allows for obtaining higher amounts of isoamyl acetate compared to *Saccharomyces* strains [39]. Our studies showed the opposite tendency (Table 5). Our previous research [65] concerned the physicochemical characterization of wines produced using indigenous yeasts from cold climate grapes. *S. cerevisiae* strain MH020215 has been shown to produce comparable amounts of isoamyl acetate and slightly lower ethyl acetate in cold climate wines, compared to the results in this article [65]. Other esters such as ethyl hexanoate, ethyl butanoate, ethyl pyroracemate, ethyl octanoate and diethyl succinate were identified in slightly lower concentrations in the analysed samples (Table 5). The wines obtained as a result of simultaneous and sequential fermentation were characterized by higher levels of these components (Table 4), similarly as in the case of the previously mentioned compounds. The remaining esters present in the wines occurred at a relatively low level (Table 5). According to the literature data, *M. pulcherrima* produces significant amounts of esters [16], especially ethyl octanoate [57,66]. The conducted research did not confirm this fact. In the wine inoculated with *M. pulcherrima* MG970690 (Mp) monoculture, this compound was not found (Table 5).

The main carbonyl compounds that occur in wine are aldehydes, and in smaller quantities, ketones. They emerge as by-products of fermentation, but also in the process of wine aging. Their concentration can vary considerably, depending on the conditions of the wine production and storage. The content of carbonyl compounds is higher in sweet wines than in dry wines, which is probably related to the oxidation of sugars. According to the literature the most important are acetaldehyde (3–494 mg/L), acetoin (0.7–350 mg/L) and diacetyl (0.1–7.5 mg/L) [67]. The aldehydes present in grapes are involved in the creation of varietal aromas, and their amount in young wines usually does not exceed 75 mg/L. The most important compound of this group is acetaldehyde, followed by propionic, isobutyric and isovaleric aldehyde. Higher amounts of acetaldehyde were detected in beverages fermented with *S. cerevisiae* MH020215 (Sc) monoculture, as well as those obtained with the use of mixed yeast cultures (Table 5). Much higher amounts of the analysed component were found in wines fermented sequentially with *M. pulcherrima* MG970690 (Mp+Sc(3), Mp+Sc(6)), in relation to *Z. bailli* 749 (Table 5). The profiles of volatile compounds in cold climate wines were analysed in our previous studies [65]. In wines produced with the use of the *S. cerevisiae* strain MH020215, higher acetaldehyde values (27.4–33.7 mg/L) were noted compared to the results obtained in this study. Also, Li and de Orduña [68] analyzed the production of acetaldehyde in fermenting grape must by yeast strains of oenological importance. It was found that both the *S. cerevisiae* and non-*Saccharomyces* strains showed similar metabolic kinetics with the highest acetaldehyde content at the beginning of fermentation followed by its reutilization. In wines obtained with the participation of *M. pulcherrima* yeast, the amount of acetaldehyde was low (<2 mg/L), while the amount of acetaldehyde was higher for *Z. bailli* (<25 mg/L) [68]. In our research, the content of acetaldehyde in wines fermented with the monocultures of *M. pulcherrima* MG970690 and *Z. bailli* 749 did not exceed 1 mg/L (Table 5). Clemente-Jimenez et al. [66] demonstrated the synthesis of significant amounts of acetaldehyde in wine by strains of *C. stellata*, *H. uvarum*, *I. orientalis*, *I. terricola* and *M. pulcherrima* in relation to *S. cerevisiae*. Similar results were achieved by Mateos et al. [69], indicating higher levels of acetaldehyde in wines produced with native microbiota compared to *Saccharomyces* yeast. Nonenal and decanal amounts were considerably lower in wines investigated. In the aspect of analysed compounds no substantial differences were found between the wines inoculated with non-*Saccharomyces* yeast and the strains of *S. cerevisiae* MH020215 (Table 5).

The presence of carboxylic acids was also noted in the analysed wines (Table 5). Octanoic acid was present in wines fermented with the *M. pulcherrima* MG970690 monoculture. In greater amounts, this component was present in wines fermented simultaneously and sequentially (Mp+Sc, Mp+Sc (3)). The exception was the wine that was inoculated with *S. cerevisiae* MH020215 yeast on the sixth day of fermentation (Table 5). *M. pulcherrima* yeast is capable of producing acetic acid in wines, which results in a rancid and pungent odour. These yeasts also produce hexanoic acid, which gives the wines the smell of fat and cheese [51]. In our research, no presence of hexanoic acid was found in wines fermented with non-*Saccharomyces* monocultures (Table 5).

The presence of terpenes was reported in the tested wines. Larger amounts of α-terpineol were found in wine fermented with the monoculture *M. pulcherrima* MG970690 (Table 5). Significant amounts of this compound were detected in simultaneously and sequentially fermented wines (Mp+Sc, Mp+Sc (3)). A similar relationship was noted for damascenone. Interestingly, lower amounts of these components were detected during sequential fermentation in which the yeast *S. cerevisiae* MH020215 was inoculated on the sixth day of the process (Mp+Sc(6)), (Table 5). Non-*Saccharomyces* yeasts, including *M. pulcherrima*, are capable of releasing distinct aromas from precursors such as glycosylated terpenes or bonded thiols via ß-glucosidase or C-S-lyase. Non-*Saccharomyces* yeast allows obtaining freshness of wine by synthesizing and subsequently free enzymes capable of releasing volatile thiols or terpenes (geraniol, linalool) [51,70].

## 3. Materials and Methods

### 3.1. Materials

Strains of *Saccharomyces cerevisiae* MH020215, *Zygosaccharomyces bailii* 749 and *Metschnikowia pulcherrima* MG970690 from the collection of Department of Fermentation Technology and Microbiology, Faculty of Food Technology, University of Agriculture in Krakow (Poland) were used in this research. *Metschnikowia pulcherrima* MG970690 and *Saccharomyces cerevisiae* MH020215 strains used for the research were isolated from spontaneous grape fermentation [71,72,73].

#### 3.1.1. Inoculum Preparation

The three-step propagation was used to multiply cultures. In the first stage, the strains were propagated on Sabouraud agar slants (Biocorp, Warsaw, Poland) for 24 h, then were introduced to 10 mL of liquid Sabouraud broth (Biocorp). After another 24 h, dynamic propagation was conducted in 200 mL of liquid Sabouraud broth (Biocorp) for 48 h on a water bath shaker (120 rpm, 28 °C). After cultivation was completed, dry weight of yeast was determined with a moisture analyser (Radwag WPS 210S, Zakład Mechaniki Produkcyjnej, Radom, Poland), and then the adequate amount of yeast slurry was centrifuged (10 min, 4989× *g*/min). The precipitate was washed with sterile water, centrifuged again under the same conditions and introduced into the grape must.

#### 3.1.2. Grape Musts Fermentation

The raw material used for fermentation was a Leon Millot grape juice, obtained by pressing the grapes using a basket press. The juice was poured into 4-L bottles and pasteurized. The total acidity of the must was set to 10 g/L, using l-malic acid. Each of 500 mL flasks was filled with 300 mL of juice. The yeast slurry was introduced into the batch in the amount of 0.5 g d.w/L. After closing the fermentation flasks and fixing fermentation tubes filled with glycerin, the setup was additionally sealed with a parafilm. The fermentation process was carried out for 28 days at 20 °C.

The samples were inoculated in three repetitions, according to the following scheme:

Monocultures: *S. cerevisiae* MH020215 (0.5 g d.w./L; Sc), *Z. bailii* 749 (0.5 g d.w./L; Zb), *M. pulcherrima* MG970690 (0.5 g d.w./L; Mp),

Simultaneous fermentation: *S. cerevisiae* MH020215 (day zero; 0.4 g d.w./L) + *Z. bailii* 749 (day zero; 0.1 g d.w./L; Zb+Sc); *S. cerevisiae* MH020215 (day zero; 0.4 g d.w./L) + *M. pulcherrima* MG970690 (day zero; 0.1 g d.w./L; Mp+Sc),

Sequential fermentation: *Z. bailii* 749 (day zero; 0.1 g d.w./L) + *S.cerevisiae* MH020215 (day three; 0.4 g d.w/L; Zb+Sc(3)), *M. pulcherrima* MG970690 (day zero; 0.1 g d.w./L) + *S. cerevisiae* MH020215 (day three; 0.4 g d.w/L; Mp+Sc(3)), *Z. bailii* 749 (day zero; 0.1 g d.w./L) + *S. cerevisiae* MH020215 (day six; 0.4 g d.w/L; Zb+Sc(6)), *M. pulcherrima* MG970690 (day zero; 0.1 g d.w./L) + *S. cerevisiae* MH020215 (day six; 0.4 g d.w/L; Mp+Sc(6)).

### 3.2. Analytical Methods

#### 3.2.1. Determination of Fermentation Dynamics

Fermentation dynamics were determined based on the weight losses of the batches during the process. Weight losses were measured daily. The process was completed at the moment of achieving daily weight losses below 0.01 g/L.

#### 3.2.2. Determination of Biomass Growth Yield

Biomass after centrifugation was washed with distilled water, and then dried in a moisture analyzer to a constant mass. Yeast dry mass was expressed in g/L.

#### 3.2.3. Determination of Ethyl Alcohol Content, Real Extract, Total Acidity and Volatile Acidity

The analysis was performed in accordance with the official International Organisation of Vine and Wine methodology (OIV 2012) [32]. The alcohol concentration in finished wine was determined using the pycnometric method. For this purpose, the distillation of samples after fermentation was performed. The obtained distillate was filled up to 100 mL with distilled water, its density was determined, and the concentration of ethanol was obtained from the adequate tables.

In order to determine real extract content, distillation residues were quantitatively transferred to a 100 mL volumetric flask, filled up to 100 mL using distilled water and the procedure was analogous.

The potentiometric method was applied to determine total acidity, titrating a sample with 0.1 M NaOH solution to obtain pH = 7. Volatile acids were separated from the wine by steam distillation and titrated using standard sodium hydroxide solution.

#### 3.2.4. Determination of Nitrogen Compounds

To determine free amino nitrogen (FAN) content, the samples were diluted in distilled water and 2 mL of diluted samples were transferred to glass tubes using a pipette. 1 mL of colour ninhydrin reagent was added, followed by boiling for 16 min in a boiling water bath. After cooling, 5 mL of the diluting agent was added into the tubes and the absorbance was measured at a wavelength of λ = 575 nm. A glycine solution containing 2 mg/L of nitrogen was used as a standard solution. The result was calculated using formulas to determine the amount of nitrogen in the sample:

$$nitrogen\;content=\frac{\mathrm{sample}\;\mathrm{absorption}\;\cdot \;2\;\mathrm{mg}\;\mathrm{of}\;\mathrm{nitrogen}}{\mathrm{standard}\;\mathrm{solution}\;\mathrm{absorption}}\cdot 50$$

#### 3.2.5. Determination of Volatile Compounds (SPME-GC-MS)

In order to determine the headspace volatile compounds, 1 g of NaCl and a 2 mL sample of must/wine were placed into a 10 mL vial. Next, an internal standard solution was added (0.57 mg/L 4-methyl-2-pentanol, 0.2 mg/L anethol and 1.48 mg/L ethyl nonanoate, Sigma-Aldrich, St. Louis, MO, USA). The SPME device (Supelco Inc., Bellefonte, PA, USA) coated with PDMS (100 μm) fiber was first conditioned by inserting it into the gas chromatograph injector port at 250 °C for 1 h. For sampling, the fiber was inserted into the headspace under stirring (300 rpm) for 30 min at 60 °C. Subsequently, the SPME device was introduced into the injector port of a 7890B chromatography system (Agilent Technologies) equipped with a Pegasus High Throughput TOFMS (LECO,) and kept in the inlet for 3 min. The SPME process was automated using the MultiPurpose Sampler (MPS, GERSTEL, Linthicum, WA, USA). The tested components were separated on a Rtx-1ms capillary column (Crossbond 100% dimethyl polysiloxane, 30 m × 0.53 mm × 0.5 μm). The detector was 250 °C, and the column was heated using the following temperature program: 40 °C for 3 min at an increment of 8 °C/min to 230 °C, then maintaining a constant temperature for 9 min. Carrier: Helium at 1.0 mL/min constant flow. EIMS electron energy 70 eV; ion source temperature and connection parts: 250 °C. The analyte transfer was performed in splitless mode; the MSD was set to scan mode from m/z = 40 to m/z = 400. Compounds were identified using mass spectral libraries and linear retention indices, calculated based on a series of n-alkanes from C6 to C30. The qualitative and quantitative identification of volatile substances (ethyl acetate, ethyl butanoate, isoamyl acetate, ethyl hexanoate, ethyl octanoate, 2-phenylethyl acetate, ethyl decanoate, ethyl dodecanoate, ethyl tetradecanoate, ethyl hexadecanoate, ethyl octadecanoate, 2-methyl-1-propanol, 2-methyl-1-butanol, 1-hexanol, 3-methyl-1-butanol, 2-phenylethanol, 1- nonanol, 1-decanol, hexanoic acid, octanoic acid, n-decanoic acid, α-terpineol, ß-damascenone, decanal; Sigma-Aldrich, St. Louis, MO, USA) were based on a comparison of retention times and peak surface area reads from samples and standard chromatograms. Other detected components were determined semiquantitatively (μg/L) by measuring the relative peak area of each identified compound according to the National Institute of Standards and Technology (NIST) database in relation to that of the internal standard. Each of the tests were performed three times.

### 3.3. Statistical Analysis

The results are presented as the means of three independent repetitions with the determination of the standard deviation. Additionally, one-way analysis of variance (ANOVA) and a test of the difference between means (Duncan’s test) were also performed (StatSoft Poland, Cracow, Poland). A heat map was prepared using the statistical package SPSS 18.0 (SPSS Inc., Chicago, IL, USA).

## 4. Conclusions

The fermentation dynamics and analytical profiles of the obtained wines showed that the use of mixed yeast cultures contributed to the reduction of volatile acidity and acetic acid content in the wines, as well as obtaining a favorable aroma profile of the wines. The yeast *Z. bailli* 749 showed very favorable deacidifying properties. Sequential fermentation resulted in a greater reduction of total acidity in the obtained wines, as compared to the simultaneous fermentation. The dominant higher alcohols in all wines were 2-methyl-1-propanol, 3-methyl-1-butanol and 2-methyl-1-butanol. Significantly higher amounts of the first two compounds were found in wines obtained with *M. pulcherrima* MG070690, both in monoculture and in mixed cultures. The monocultures of *M. pulcherrima* MG070690 (Mp) compared with *Z. bailli* 749 (Zb) synthesized higher levels of esters in wines, including ethyl acetate, ethyl propionate, isobutyl acetate, ethyl pyroracemate and isoamyl acetate. Octanoic acid was present in wines fermented with the *M. pulcherrima* MG970690 monoculture. In greater amounts, this component was present in wines fermented simultaneously and sequentially (Mp+Sc, Mp+Sc (3)). A similar relationship were noted for α-terpineol and damascenone A. Mixed fermentations of non-*Saccharomyces* yeasts in combination with *S. cerevisiae* can therefore be used as a tool to modulate flavour profiles and improve oenological parameters of wines.

## Figures and Tables

**Table 1:** Characterization of fresh grape must (mean of 3 series ± standard deviation).

Grape Must	Total Acidity * [g/L]	FAN [mg/L]	Citric Acid [g/L]	Tartaric Acid [g/L]	l-Malic Acid [g/L]	Succinic Acid [g/L]	Lactic Acid [g/L]	Acetic Acid [g/L]
	10.00 ** (±0.00)	106 (±5.6)	0.06 (±0.00)	2.54 (±0.21)	3.89 (±0.20)	0.94 (±0.07)	0.00 (±0.00)	0.00 (±0.00)

* expressed in g/L of malic acid. ** after acidification with l-malic acid.

**Table 2:** Characterization of analysed wines (mean of 3 series ± standard deviation).

Yeast/Parameters of Wine	Biomass [g/L]	Total Acidity * [g/L]	Volatile Acidity ** [g/L]	Ethanol [*v/v*]	Extract [g/L]	FAN [mg/L]
*S. cerevisiae* MH020215 (Sc)	0.88 a (±0.11)	9.29 abc (±0.37)	0.19 a (±0.05)	9.89 a (±0.12)	23.2 a (±0.00)	14.6 ab (±2.3)
*Z. bailli* 749 (Zb)	1.16 a (±0.24)	9.00 bc (±0.1)	0.25 ab (±0.05)	4.25 c (±2.57)	117 c (±4.4)	83.6 d (±3.5)
*M. pulcherrima* MG970690 (Mp)	1.17 a (±0.21)	10.1 a (±0.67)	0.30 c (±0.07)	6.82 b (±2.5)	73.4 b (±4.4)	77.2 d (±2.5)
*Z. bailli* 749 + *S. cerevisiae* MH020215 (Zb+Sc)	1.02 a (±0.12)	9.62 abc (±0.43)	0.16 a (±0.03)	9.73 a (±0.17)	24.1 a (±1.5)	16.4 abc (±3.5)
*M. pulcherrima* MG970690 + *S. cerevisiae* MH020215 (Mp+Sc)	1.15 a (±0.22)	10.1 a (±0.68)	0.16 a (±0.02)	9.79 a (±0.33)	24.1 a (±1.5)	13.0 a (±0.8)
*Z. bailli* 749 + *S. cerevisiae* MH020215 (Zb+Sc(3))	1.03 a (±0.18)	9.29 abc (±0.5)	0.21 a (±0.08)	9.37 a (±0.75)	22.3 a (±1.5)	13.7 ab (±0.1)
*M. pulcherrima* MG970690 + *S. cerevisiae* MH020215 (Mp+Sc(3))	0.85 a (±0.17)	10.1 a (±0.61)	0.18 a (±0.03)	9.10 ab (±0.21)	20.6 a (±0.00)	20.8 bc (±2.7)
*Z. bailli* 749 + *S. cerevisiae* MH020215 (Zb+Sc(6))	1.00 a (±0.26)	8.77 b (±0.12)	0.18 a (±0.03)	8.66 ab (±0.4)	23.2 a (±0.00)	35.9 e (±2.6)
*M. pulcherrima* MG970690 + *S. cerevisiae* MH020215 (Mp+Sc(6))	1.71 b (±0.71)	9.89 ac (±0.57)	0.16 a (±0.02)	8.63 ab (±0.26)	25.8 a (±0.00)	23.2 c (±2.7)

* expressed in g/L of malic acid; ** expressed in g/L of acetic acid. The mean values marked with different letters in the columns show differentiation according to Duncan’s test (*p* < 0.05).

**Table 3:** Characterization of organic acids profiles in obtained wines (mean of 3 series ± standard deviation).

Yeast/Parameters of Wine	Citric Acid [g/L]	Tartaric Acid [g/L]	l-Malic Acid [g/L]	Succinic Acid [g/L]	Lactic Acid [g/L]	Acetic Acid [g/L]
*S. cerevisiae* MH020215 (Sc)	0.07 a (±0.00)	2.60 (±0.55)	3.20 abc (±0.39)	1.62 ab (±0.15)	0.00 a (±0.00)	0.02 a (±0.02)
*Z. bailli* 749 (Zb)	0.08 a (±0.01)	2.25 (±0.54)	3.62 c (±0.12)	0.83 e (±0.05)	2.30 c (±0.33)	0.06 ab (±0.03)
*M. pulcherrima* MG970690 (Mp)	0.13 b (±0.06)	2.23 (±0.31)	3.57 bc (±0.33)	1.16 d (±0.29)	1.61 b (±0.04)	0.09 b (±0.05)
*Z. bailli* 749 + *S. cerevisiae* MH020215 (Zb+Sc)	0.07 a (±0.01)	2.33 (±0.38)	3.36 abc (±0.06)	1.71 b (±0.02)	0.00 a (±0.00)	0.00 a (±0.00)
*M. pulcherrima* MG970690 + *S. cerevisiae* MH020215 (Mp+Sc)	0.06 a (±0.01)	2.12 (±0.03)	3.26 abc (±0.30)	1.67 ab (±0.06)	0.00 a (±0.00)	0.02 a (±0.02)
*Z. bailli* 749 + *S. cerevisiae* MH020215 (Zb+Sc(3))	0.07 a (±0.01)	2.10 (±0.26)	3.06 ab (±0.49)	1.47 abc (±0.26)	0.00 a (±0.00)	0.04 ab (±0.04)
*M. pulcherrima* MG970690 + *S. cerevisiae* MH020215 (Mp+Sc(3))	0.07 a (±0.00)	2.20 (±0.54)	3.00 a (±0.20)	1.32 cd (±0.12)	0.00 a (±0.00)	0.02 a (±0.00)
*Z. bailli* 749 + *S. cerevisiae* MH020215 (Zb+Sc(6))	0.07 a (±0.00)	2.86 (±0.13)	3.24 abc (±0.19)	1.42 acd (±0.03)	0.00 a (±0.00)	0.02 a (±0.01)
*M. pulcherrima* MG970690 + *S. cerevisiae* MH020215 (Mp+Sc(6))	0.06 a (±0.00)	2.17 (±0.72)	3.06 ab (±0.04)	1.49 abc (±0.06)	0.00 a (±0.00)	0.01 a (±0.00)

The mean values marked with different letters in the columns show differentiation according to Duncan’s test (*p* < 0.05).

**Table 4:** Characterization of volatile compounds of grape must (mean of three series ± standard deviation).

Compound	LRI ^2^	[µg/L]
Hexanal	800	39.3 (±4.89)
2-Hexenal ^3^	853	22.3 (±3.50)
3-Hexen-1-ol	858	82.3 (±6.81)
2-Hexen-1-ol	872	7.41 (±1.14)
1-Hexanol	880	714 (±26.41)
2-Heptenal ^3^	958	37.4 (±6.73)
1-Octen-3-one	988	16.5 (±2.30)
1-Octen-3-ol	999	26.3 (±4.05)
2-Pentylfuran ^3^	1010	5.01 (±1.30)
2-Ethyl-1-hexanol ^3^	1020	43.3 (±6.06)
2-Octenal ^3^	1049	8.79 (±0.84)
2-Octen-1-ol ^3^	1066	7.97 (±0.30)
Nonanal	1102	42.6 (±3.45)
1-Nonanol	1156	57.3 (±8.60)
Decanal	1182	45.2 (±6.33)
2-[(2-Ethylhexyl)oxy]-ethanol ^3^	1226	282 (±25.06)
2-Decenal ^3^	1250	1.91 (±0.27)
1-Decanol	1272	208 (±17.32)
2-Undecenal ^3^	1350	3.99 (±0.57)
1-Undecanol	1374	244 (±27.32)
β-Damascenone	1384	24.5 (±8.59)
2-Dodecanol ^3^	1417	5.62 (±0.80)
Geranylacetone	1453	32.6 (±1.95)
*trans*-β-Ionone	1485	0.63 (±0.13)
2,4-Di-*tert*-butylphenol ^3^	1490	7.44 (±0.10)

^2^ LRI—Linear Retention Index. ^3^ determined semi-quantitatively by measuring the relative peak area of each identified compound, according to the NIST database, in relation to that of the internal standard.

**Table 5:** A heat map analysis of 49 volatiles [μg L^−1^] in wines produced using mono- and mixed cultures of yeasts (mean of three series). The highest content is in the darkest green and the lowest content is in the darkest red.

Compound [µg/L]	LRI^2^	*S. cerevisiae* MH020215	*Z. bailli* 749	*M. pulcherrima* MG970690	*Z. bailli* 749 + *S. cerevisiae* MH020215	*M. pulcherrima* MG970690 *+ S. cerevisiae* MH020215	*Z. bailli* 749+ *S. cerevisiae* MH020215 (3)	*M. pulcherrima* MG970690+ *S. cerevisiae* MH020215 (3)	*Z. bailli* 749 + *S. cerevisiae* MH020215 (6)	*M. pulcherrima* MG970690 + *S. cerevisiae* MH020215 (6)
**Esters**										
Ethyl acetate	614	33921 a	8753 e	36483 ab	65972 c	41459 ab	46897 ab	133551 f	51376 bc	34026 a
Ethyl propionate	714	5269 c	190 b	196 b	5116 c	7150 d	2424 a	7513 d	2888 a	2266 a
Isobutyl acetate	771	16.3 a	18.2 a	191.4 c	17.7 a	34.9 a	33.6 a	97.5 b	44.9 a	41.4 a
Ethyl pyroracemate ^3^	785	228 b	24 a	197 b	645 e	0 a	419 c	1543 g	544 d	897 f
Ethyl butanoate	789	153 c	0 a	11 a	135 bc	141 bc	97 bd	258 e	76 d	27 a
2-Hydroxyethyl propionate ^3^	798	99 a	0 b	1 b	96 a	468 c	135 a	338 d	102 a	520 c
Ethyl 2-ethyl-3-methylbutanoate ^3^	847	12.9 b	0.0 a	0.0 a	0.0 a	0.0 a	0.0 a	0.0 a	0.0 a	0.0 a
Ethyl 3-methylbutanoate	849	11.9 c	0.0 a	0.0 a	6.7 b	0.0 a	0.0 a	0.0 a	0.0 a	0.0 a
Isoamyl acetate	884	1415 b	85 a	137 a	1596 b	300 a	673 c	280 a	270 a	110 a
2-Methylbutyl acetate	886	0.0 a	12.4 b	0.0 a	0.0 a	0.0 a	0.0 a	0.0 a	0.0 a	65.7 c
Ethyl hexanoate	986	257.8 de	7.7 a	3.2 a	295.6 e	230.7 d	174.1 c	467.9 f	147 bc	116.9 b
Diethyl succinate	1149	953.2 ab	0.0 c	16.6 c	1707.6 d	1450.7 bd	774.8 a	2864 e	1174.4 ab	3667.0 f
Ethyl octanoate	1180	536.7 de	8.0 a	0.0 a	661.8 f	559.3 ef	396.2 bc	1058.4 g	438.4 cd	292.9 b
2-Phenylethyl acetate	1228	17.7 a	5.0 a	38.1 ab	54.2 bc	40.0 ab	201.2 e	84.2 cd	398.3 f	33431.6 g
Propyl nonanoate ^3^	1390	1.01 b	1.28 c	0.98 b	0.00 a	0.00 a	0.00 a	0.00 a	0.00 a	0.00 a
Ethyl decanoate	1397	36.6 e	2.1 c	1.6 c	95.8 ab	124.9 b	167.9 d	87.7 a	199.2 d	96.6 ab
Ethyl isopentyl succinate ^3^	1430	2.0 a	0.0 a	0.0 a	9.5 ac	21.2 b	24.9 b	17.9 bc	35.8 d	85.4 e
Ethyl dodecanoate	1581	67.4 b	1.4 b	1.2 b	229.7 ac	269.4 ac	193.6 a	194.2 a	581.9 d	322.5 c
Isopentyl decanoate ^3^	1653	3.0 a	0.0 a	0.0 a	9.8 b	16.4 c	12.9 bc	11.5 bc	25.8 d	25.4 d
Ethyl tetradecanoate	1790	17.3 ac	0.9 a	4.3 a	49.2 abc	67.1 bc	137.2 d	74.4 b	391.6 f	83.0 b
Ethyl *E*-11-hexadecenoate ^3^	1974	0.7 a	0.0 a	0.0 a	5.1 a	10.8 ab	41.2 b	7.0 ab	21.0 ab	10.8 ab
Ethyl hexadecanoate	1990	69.8 a	2.6 a	0.0 a	209.2 a	455.2 c	1120.7 d	802.3 b	1369.3 e	661.2 b
Methyl linoleate ^3^	2092	1.8 a	0.0 a	0.0 a	9.6 b	11.8 bc	21.1 d	14.2 c	43.3 f	31.6 e
Ethyl octadecanoate	2189	1.8 ab	0.0 a	0.0 a	4.4 b	9.9 e	30.8 d	15.8 c	34.2 d	15.5 c
**Alcohols**										
2-Methyl-1-propanol	617	26200 b	70261 a	182815 d	53733 ab	71599 a	79316 a	224949 e	145161 c	326167 f
3-Methyl-1-butanol	734	18954 ab	4215 a	28835 ab	43757 b	158869 d	119238 c	118715 c	112660 c	181879 d
2-Methyl-1-butanol	740	20717a	4871 c	10249 c	33176 b	53604 d	28710 ab	52710 d	22126 a	27322 ab
Isohexanol ^3^	838	20.4 ab	0.0 c	0.0 c	15.2 a	25.9 b	35.5 d	78.0 e	14.4 a	21.6 ab
3-Methylpentanol ^3^	843	132 ad	0 c	0 c	153 ab	198 b	409 e	88 d	143 a	146 a
3-Hexen-1-ol	858	21.4 ab	40.4 a	43.0 a	0.0 b	23.6 ab	37.2 a	92.5 c	25.2 ab	39.0 a
1-Hexanol	880	431 a	407 a	786 bc	531 ab	714 b	654 ab	1498 d	609 ab	968 c
2-Ethyl-1-hexanol ^3^	1020	33.2 ab	59.7 c	46.6 ac	37.0 ab	44.7 a	43.2 ab	112.0 d	29.9 b	47.1 ac
Phenylethanol	1114	9248 a	1745 b	2074 b	15516 d	24082 c	11098 a	31932 e	11071 a	27422 c
1-Nonanol	1156	16.8 b	60.9 d	46.3 c	24.6 b	0.0 a	0.0 a	0.0 a	0.0 a	0.0 a
2-[(2-Ethylhexyl)oxy]-ethanol ^3^	1226	4.3 a	38.5 c	7.3 ab	19.0 ab	23.3 bc	80.7d	117.7 f	98.0 e	94.7 de
1-Decanol	1272	2.8 a	33.8 de	5.7 a	26.6 cd	12.7 ac	60.4 bf	44.7 be	75.3 f	57.4 b
1-Undecanol	1374	1.2 b	20.0 c	4.0 b	11.3 bc	16.4 c	55.0 a	51.7 a	57.0 a	55.6 a
2-Dodecanol ^3^	1417	2.50 c	1.55 b	5.94 d	1.06 b	0.00 a	0.00 a	0.00 a	0.00 a	0.00 a
1-Dodecanol	1448	0.4 ab	2.8 a	2.4 ab	0.0 b	3.0 a	15.3 d	13.0 cd	11.9 c	12.1 c
2,4-Di-*tert*-butylphenol ^3^	1490	2.0 a	6.1 a	6.5 a	5.1 a	7.6 a	30.2 b	29.9 b	28.6 b	14.4 c
**Acetals**										
Acetaldehyde	725	1227 ab	40 a	132 a	2448 b	2070 ab	1671 ab	13696 e	7226 c	11068 d
Nonanal	1102	25.2 a	23.4 a	25.7 a	25.9 a	53.0 b	50.1 b	0.0 c	30.5 a	68.3 d
Decanal	1182	13.3 ab	20.8 ce	16.9 bc	25.8 f	11.6 ad	7.5 d	22.8 ef	16.1 abc	31.5 g
**Carboxylic acids**										
Hexanoic acid	982	154 b	0 a	0 a	0 a	239 b	484 c	1802 e	750 d	572 c
Octanoic acid	1160	610 d	0 a	5 a	347 c	883 b	1983 e	5292 g	2965 f	927 b
*n*-Decanoic acid	1371	0 a	0 a	0 a	0 a	25 b	314 c	96 b	444 d	0 a
**Terpenes**										
α-Terpineol	1171	13.8 abc	10.1 bc	14.6 ab	16.1 ab	18.2 a	18.3 a	30.9 d	14.8 ab	8.1 c
Damascenone	1384	5.0 a	9.2 ab	6.1 a	13.0 ab	16.4 b	45.5 c	44.6 c	53.5 e	32.9 d
2,6-di-*tert*-Butyl-*p*-benzoquinone ^3^	1459	0.46 a	0.78 ab	0.85 ab	1.66 ab	2.62 b	8.88 d	5.94 c	8.10 d	4.97 c

^1^ Values with different letters (a–g) in the same row are significantly different, according to the Duncan test (*p* < 0.05); ^2^ LRI—Linear Retention Index; ^3^ determined semi-quantitatively by measuring the relative peak area of each identified compound, according to the NIST database, in relation to that of the internal standard.

## Data Availability

Not applicable.

## Sample Availability

Samples of the compounds are not available from the authors.
